# Accelerating Vaccine Adjuvant Screening: Early Follicular Dendritic Cell and Germinal Center B Cell Biomarkers Predict Protective Efficacy

**DOI:** 10.3390/vaccines13101011

**Published:** 2025-09-28

**Authors:** Yiwei Zhong, Mingyue Chen, Hongzhe Lin, Zhenrui Liu, Shijie Zhang, Yue He, Bin Wang

**Affiliations:** 1 MOE/NHC/CAMS Key Laboratory of Medical Molecular Virology, Shanghai Institute of Infectious Disease and Biosecurity, Shanghai Frontiers Science Center of Pathogenic Microorganisms and Infection, School of Basic Medical Sciences, Shanghai Medical College, Fudan University, Shanghai 200032, China; yiweizhong@fudan.edu.cn (Y.Z.); 23211020003@m.fudan.edu.cn (M.C.); 21111010014@m.fudan.edu.cn (H.L.); 16621563154@163.com (Z.L.); 23111010038@m.fudan.edu.cn (S.Z.); 22111010031@m.fudan.edu.cn (Y.H.); 2 Advaccine Biopharmaceuticals (Suzhou) Co., Ltd., Suzhou 215000, China; 3 Children’s Hospital of Fudan University, Shanghai 201102, China

**Keywords:** adjuvants, germinal center B cell, follicular dendritic cell, quick adjuvant assessment, humoral immunity, vaccine, protection efficacy

## Abstract

**Background**: The current assessment method of the protective efficacy of adjuvanted vaccines remains slow and labor-intensive, hindered by prolonged immunization protocols and complex assays. **Methods:** To overcome this bottleneck, we demonstrate that early segregated cellular biomarkers enable rapid prediction of protection, using a respiratory syncytial virus (RSV) pre-fusion F (pre-F) protein model with diverse adjuvants in mice. **Results**: We identified that germinal center (GC) B cell responses (Days 7 and 9 post-immunization) strongly aligned with protective efficacy, except for Alum, which achieved MF59-level protection despite lower GC responses. Crucially, follicular dendritic cell (FDC) abundance at day 7 universally predicted protection across all adjuvants, including Alum, drastically shortening discovery time and effort from at least 4–6 weeks to within 1 week. **Conclusions:** FDCs and GC B cells serve as complementary early biomarkers that accurately forecast vaccine efficacy. This approach could potentially reduce the need for prolonged immunization regimens by cellular profiling on days 7–9, offering a modest step toward streamlining adjuvant selection and informing vaccine design.

## 1. Introduction

Vaccine adjuvants critically enhance immunogenicity, with aluminum (Alum) dominating clinical use for nearly a century. Alum elevates antibody titers in vaccines against hepatitis B, COVID-19, and diphtheria-tetanus-pertussis (DTP), primarily through Th2-biased responses [1,2]. Emulsion adjuvants like MF59 improve influenza vaccine efficacy in the elderly by promoting humoral immunity and antigen persistence [3]. Despite their utility, classical adjuvants exhibit limitations; they often fail to induce long-term protection and perform suboptimally in aged populations [4,5]. Toll-like receptor (TLR) agonists including CpG (TLR9), R848 (TLR7/8) [6], and STING agonists cGAMP show potential but require formulation optimization [7]. For instance, CpG ODN enhances Th1 responses and cellular immunity when compared to Alum, while AS04 (MPL-Alum) generates stronger antibodies and more balanced Th1/Th2 profiles in HPV/HBV vaccines [8,9]. Nevertheless, their efficacy remains inferior to Alum in humoral response amplification. Nanotechnology (e.g., lipid nanoparticles) and combinatorial systems (e.g., AS01, AS04) offer promising strategies to overcome these constraints [10,11].

Critically, the development of next-generation vaccine adjuvants is constrained by inefficient evaluation methods [12,13]. Current preclinical screening of candidates relies on time-intensive endpoints like neutralizing antibody assays (4–8 week/cycle) and in vivo viral challenges, extending timelines to nearly one year [14,15]. This delay impedes rapid responses to emerging threats (e.g., X-virus), creating an urgent need for early biomarkers that rapidly predict protective efficacy. Such biomarkers would enable the development of high-throughput in vivo platforms for accelerated adjuvant discovery against swiftly evolving pathogens.

Humoral immunity hinges on germinal center (GC) reactions where T follicular helper (Tfh) cells activate B cells [16,17]. Tfh cell priming requires antigen presentation by dendritic cells (DCs), macrophages, and monocytes in draining lymph nodes (dLNs) [18,19]. GC B cells undergo division, somatic hypermutation (SHM), and evolutionary selection. GC B cells compete for GC-Tfh help for division. Subsequently, they migrate into a light zone of GC, where follicular dendritic cells (FDCs) organize a structured network and supply antigens to GC B cells for selection. Notably, FDC-specialized stromal cells retain antigens as immune complexes (ICs) for weeks to months, enabling sustained B cell affinity maturation in GCs [20,21,22]. FDC depletion severely impairs GC responses [23], and their networks expand for a period after immunization (e.g., with adjuvants like alum or CFA) [24] We hypothesize that early quantification of FDC density or GC B cell responses in dLNs could serve as segregated biomarkers to predict the humoral immunity magnitude and quality, dramatically shortening the adjuvant evaluation timeline.

To address the critical bottleneck in adjuvant screening, we leveraged early cellular biomarkers to predict protection in mice immunized with RSV pre-F protein combined with MF59, Alum, CpG, or R848. Our data establish that innate cell activation and Tfh cell responses fail to robustly predict vaccine-induced protection. While germinal center (GC) B cell responses at days 7 and 9 post-immunization aligned with protective efficacy for most adjuvants, the Alum adjuvant achieved MF-59 level protection despite weaker GC B cell responses, revealing a key gap in prediction. Crucially, follicular dendritic cell (FDC) abundance at day 7 universally predicted protection across all adjuvants, including Alum. These complementary early biomarkers enable rapid, accurate vaccine efficacy prediction, replacing months-long immunization with cellular profiling at days 7–9 to accelerate preclinical adjuvant screening and rational vaccine design.

## 2. Materials and Methods

### 2.1. Mice, Virus, and Infection

Female 8- to 10-week-old Balb/c mice were used for all experiments and purchased from Vital River Laboratory Animal Technology Co., Ltd. (Shanghai, China). All mice were maintained under specific pathogen-free conditions at Fudan University and treated according to the animal welfare guidelines for laboratory animals (No. 20230301-038). The protocols used were approved by the Institutional Animal Care and Use Committee of Fudan University.

Plaque-purified human RSV (type A2 strain from the American Type Culture Collection, Rockville, MD, USA) was grown in HEp-2 cells and concentrated by ultracentrifugation (50,000× *g* for 1 h). Mice were infected intranasally (i.n.) with 5 × 10^7^ PFU RSV in 50 μL under anesthesia on day 14 after the second immunization.

### 2.2. Immunization

Balb/c mice were immunized with various vaccines (as detailed in Table 1), and immune cell responses were analyzed at multiple time points after immunization. For the IgG and neutralizing antibody assessments, Balb/c mice (8–10 weeks old, female) were subjected to prime–boost immunization, with the prime administered on day 0 and the boost on day 14. The mice were randomly divided into the following formulations (n = 5/group/experiment): MF59 (50 μL) (Cat# ADV810, Advaccine Biopharmaceuticals Co., Ltd., Suzhou, China) combined with pre-F (10 μg) (Cat# NP4A-1, Advaccine Biopharmaceuticals Co., Ltd., Suzhou, China); Alum (100 μg) (Cat# vac-alu-250, Invivogen, San Diego, CA, USA) combined with pre-F (10 μg); CpG ODN 1018 (20 μg) (Advaccine Biopharmaceuticals Co., Ltd., Suzhou, China) combined with pre-F (10 μg); and R848 (20 μg) (Advaccine Biopharmaceuticals Co., Ltd., Suzhou, China) combined with pre-F (10 μg). Pre-F (10 μg) alone served as the antigen control; PBS served as the vehicle control (Table 1). To assess the kinetics of the T follicular helper (Tfh), germinal center (GC) B cell, and plasma cell responses, the immunized mice were euthanized on days 5, 7, and 9 following a single immunization. For profiling the innate immune response, the immunized mice were euthanized on days 1 and 2 post-immunization. Additionally, to profile FDC response, the immunized mice were euthanized on days 3, 5, 7, and 9 post-immunization.

### 2.3. Enzyme-Linked Immunosorbent Assay (ELISA)

The serum was collected on day 14 and day 28 post-prime for IgG endpoint titer measurement by ELISA. ELISA was used to quantify RSV-specific IgG, IgG1, and IgG2a titers in serum, as previously described [25]. Briefly, 96-well plates (Corning, New York, NY, USA) were coated with pre-F protein (2 μg/mL) (50 mM carbonate bicarbonate buffer, pH 9.6) at 37 °C and blocked with 5% BSA in PBST (0.05% Tween 20 in PBS) at 37 °C. Plates were incubated with serial 4-fold dilutions of serum for 1 h at 37 °C. HRP-conjugated secondary antibodies (Southern Biotech, Birmingham, AL, USA) were applied and incubated for 45 min at 37 °C.

### 2.4. Viral Neutralizing Antibody Assay

The neutralizing antibody assay was performed as previously described [26]. Serum was collected on day 28 post-prime for neutralizing antibody quantification assays. Serum samples were serially diluted 5-fold in PBS, heat-inactivated at 56 °C for 30 min, and incubated with a 3 × 10^3^ 50% tissue culture-infective dose of virus for 2 h at 4 °C. HEp-2 cells (ATCC CCL-23) were added to each well. Plates were incubated for 3 days in a 5% CO_2_ incubator at 37 °C, fixed with 80% cold acetone in PBS, and blocked with 3% blocking buffer. Goat anti-RSV antibody (Meridian Life Science, Saco, ME, USA) was added, and the enzymatic reaction was developed. Optical densities were read at 450/620 nm using an ELISA microplate reader (Thermo Fisher Scientific, Waltham, MA, USA). Neutralizing antibody titers were calculated using the Reed–Muench method.

### 2.5. Draining Lymph Node Isolation and Flow Cytometry

The draining lymph nodes were dissected from the immunized mice and homogenized through a 40 µm strainer. The single-cell suspensions in dLN were stained for 15 min with a viability marker (LiveDead-efluor780, eBioscience, San Diego, CA, USA) and with the following antibodies. For analysis of DCs, the following antibodies were used: anti-CD11c-BV421 (N418, Biolegend, San Diego, CA, USA) and anti-IA/IE-PerCP/cy5.5 (M5/114.15.2, Biolegend). To analyze monocytes, macrophages, neutrophils, and eosinophils, single cells were stained with the following antibodies: anti-CD11b-BV510 (M1/70, Biolegend), anti-Ly6C-FITC (HK1.4, Biolegend), anti-Ly6G-APC (1A8, Biolegend), anti-Siglec F-AF700 (1RNM44N, eBiosience, San Diego, CA, USA), anti-F4/80-PE (BM8, Biolegend), and anti-CD169-PerCP/cy5.5 (3D6.112, Biolegend). For analysis of Tfh cells, the following antibodies were used: anti-CD3-BV510 (17A2, Biolegend), anti-CXCR5-FITC (L138D7, Biolegend), anti-PD-1-PerCP/Cy5.5 (29F.1A12, Biolegend), and anti-CD4-AF700 (11B11, Biolegend). For analysis of GC B and plasma cells, the following antibodies were used: anti-B220-BV605 (RA3-6B2, Biolegend), anti-CD138-BV510 (281-2, Biolegend), anti-CD44-FITC (IM7, Biolegend), anti-CD38-PerCP-eFluor 710 (90, Invitrogen, Carlsbad, CA, USA), and anti-GL7-APC (GL7, Biolegend). For analysis of FDCs, the following antibodies were used: anti-CD31-FITC (390. Biolegend), anti-B220-PE (RA3-6B2, Invitrogen), anti-CD21/CD35-APC (7E9, Biolegend), anti-CD45-PerCP/Cy5.5 (30-F11, Biolegend), and anti-PDPN-BV421 (8.1.1, Biolgend). The absolute counts of cells were obtained using Precision Count Beads (424902, Biolegend), following the protocol provided. Flow cytometry was performed using the LSRFortessa (BD Biosciences, Franklin Lakes, NJ, USA) and data were analyzed using FlowJo software (V10.8.1) (BD Biosciences).

### 2.6. Fluorescent Labeling of Pre-F and Quantification of the Number of Pre-F-Specific GC B Cells

Biotinylated pre-F (11049-V49H-B, SinoBiological, Beijing, China) was mixed with fluorochrome-conjugated streptavidin, either BV421 streptavidin (Cat# 405226, Biolegend) or FITC streptavidin (Cat# 405201, Biolegend), at room temperature for 60 min to generate fluorescently labeled pre-F tetramer. Lymphocytes from mouse lymph nodes on days 7 and 9 post-single immunization were incubated with a 1:1 mixture of pre-F-BV421 and pre-F-FITC. Subsequently, both pre-F-BV421- and pre-F-FITC-positive cells within the GC B cells were identified. The quantification of the number of cells is detailed in the section regarding draining lymph node isolation and flow cytometry.

### 2.7. Statistical Analysis

Data are presented as means ± SEM. Statistical analysis was conducted using GraphPad Prism software (V9.5.1) (La Jolla, CA, USA). Differences in mean values between three or more groups were assessed using one-way ANOVA with Tukey’s post hoc test for multiple comparisons. Significance levels are as follows: **** *p* < 0.0001; *** *p* < 0.001; ** *p* < 0.01; * *p* < 0.05; not significant—ns. Pearson’s correlation coefficient (r) was used to quantify linear relationships between continuous variables. Data points in all figures represent independent lymph nodes, unless otherwise specified. Sample sizes (n = 3–5/group) were based on pilot data. While no pre-experimental power analysis was performed, a post hoc analysis (GraphPad Prism, α = 0.05) confirmed sufficient power for key correlations.

## 3. Results

### 3.1. Variable Vaccine Protection Is Driven by Distinct Adjuvants

**Figure 1:**
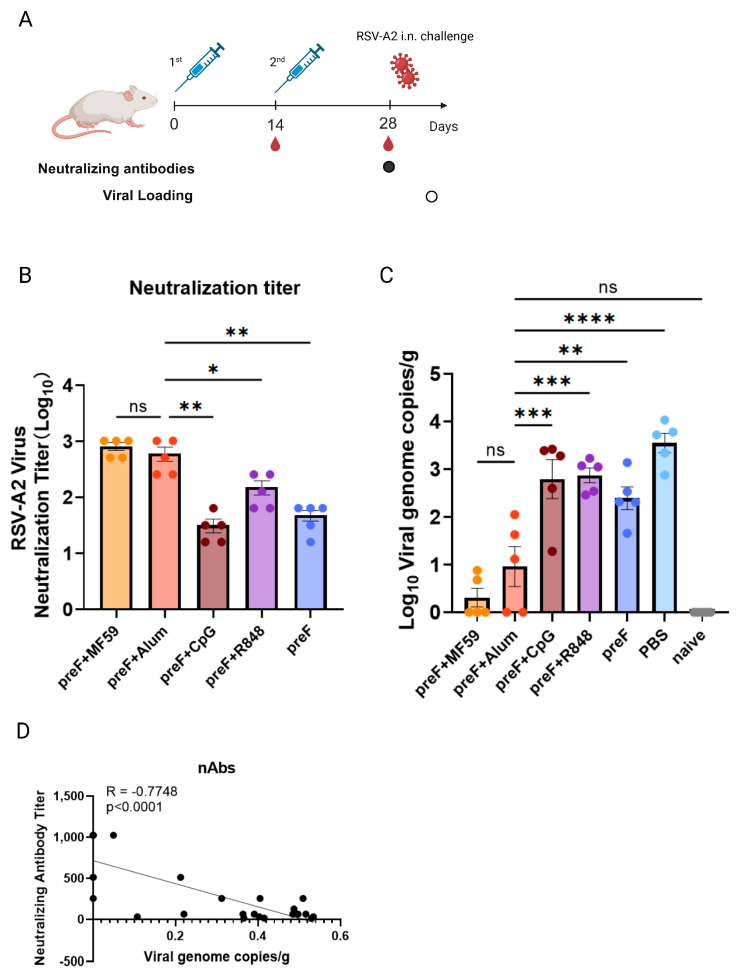
**Serum neutralizing antibody titers correlate with protection against RSV**−**A2 challenge**. (**A**) Experimental schematic for (**B**–**D**). The solid dot represents the timepoint of neutralizing antibody detection and the hollow dot represents the timepoint of lung viral loads detection. Balb/c mice (8–10 weeks old, female) were subjected to two immunizations, with the prime administered on day 0 and the boost on day 14. Serum was collected on day 28 post-prime for neutralizing antibody quantification assays. On day 29, mice were intranasally challenged with RSV-A2, and lungs were harvested for viral load quantification 4 days post-challenge. (**B**) RSV-A2-specific serum neutralizing antibody titers measured pre-challenge. (**C**) Viral genome copies per gram of lung tissue quantified by qPCR targeting the RSV N gene. (**D**) Pearson correlation between neutralizing antibody titers and lung viral loads. Representative data from two independent experiments, each with five mice per group (means ± SEM). **** *p* < 0.0001, *** *p* < 0.001, ** *p* < 0.01, * *p* < 0.05, ns (not significant) by one-way ANOVA.

To evaluate the extent of the impact of various adjuvants on protective efficacy, we used the respiratory syncytial virus (RSV) prefusion F (pre-F) protein as the antigen. Mice were subcutaneously immunized twice with RSV pre-F antigen combined with MF59, Alum, CpG1018, or R848 adjuvants, or with pre-F antigen alone (pre-F -alone), at days 0 and 14 (Figure 1A). Serum samples were collected following the second immunization to assess neutralizing antibody titers (nAb). Subsequently, mice were challenged with RSV-A2, and lung viral loads were quantified on day 5 post-challenge. The nAb titer results showed that MF59 and Alum adjuvanted groups had highest nAb titers, with no significant difference between them (*p* > 0.05). Alum+pre-F demonstrated significantly higher titers than the CpG+pre-F (*p* < 0.01), R848+pre-F (*p* < 0.05), and pre-F-alone (*p* < 0.01) groups (Figure 1B).

The protection efficacy against RSV-A2 was evaluated by lung viral load. The results showed that the MF59 and Alum adjuvanted groups conferred the strongest protection, exhibiting significantly lower viral loads than the CpG+pre-F (*p* < 0.001), R848+pre-F (*p* < 0.001), and pre-F-alone (*p* < 0.01) groups (Figure 1C). The correlation result exhibited that nAbs were a high negative correlation coefficient (r = −0.7748) with viral loads (Figure 1D). This confirms variable adjuvant efficacy but highlights the impracticality of multi-week nAb/challenge models for rapid screening. While nAbs are indicative, their longevity suggests that the need for a rapid assessment method suitable for high-throughput screening.

### 3.2. Binding Antibody Titers Lack Correlation with Protection Efficacy

**Figure 2:**
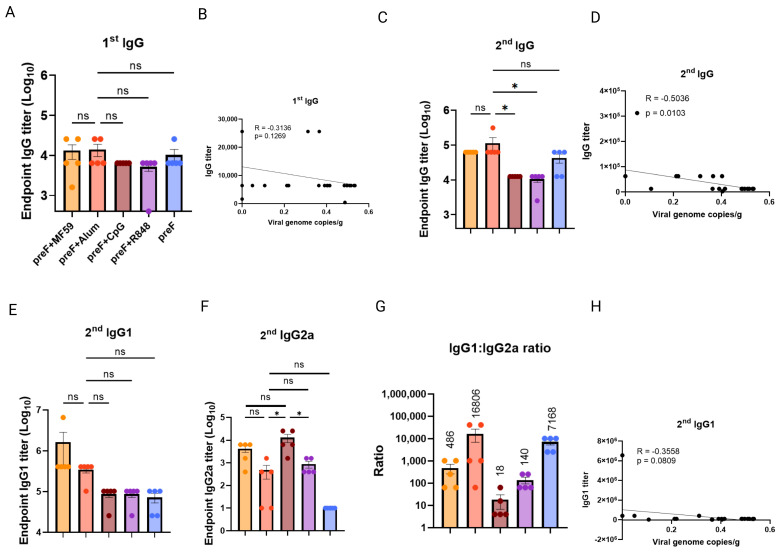
**Serum IgG endpoint titers show poor correlation with the protection.** The serum was collected on day 14 post−first and -second immunization for IgG endpoint titer measurement by ELISA. These samples were derived from the mice presented in Figure 1. The orange dots: MF59+pre−F; The red dots: Alum+pre−F; The brown dots: CpG+pre−F; The purple dots: R848+pre−F; The blue dots: pre−F alone. (**A**) Titers of pre−F−specific binding IgG were measured by endpoint dilution ELISA using sera of immunized mice collected after first immunization. (**B**) Pearson correlation between the first IgG titers and lung viral loads. (**C**) Titers of pre-F-specific binding IgG after second immunization. (**D**) Correlation between the second IgG titers and lung viral loads. Titers of pre-F-specific binding IgG1 (**E**) and IgG2a (**F**) after second immunization. (**G**) The IgG1/IgG2a ratio was analyzed. (**H**) Pearson correlation between the second IgG1 titers and lung viral loads. Representative data from two independent experiments, each with five mice per group (means ± SEM). * *p* < 0.05; ns (not significant) by one-way ANOVA.

To explore whether binding antibodies were linked with efficacy, we assessed the titer of pre-F-specific binding IgG post-first and -second immunization, as well as IgG1 and IgG2 titer post-second immunization. The mice were from Figure 1. The results showed that no significant differences in pre-F-IgG titers post-first immunization were observed among the groups immunized with pre-F combined with MF59, CpG, or R848 adjuvants compared to the Alum+pre-F group (*p* > 0.05) (Figure 2A) and IgG titer post-first immunization showed a low negative correlation coefficient (r = −0.3136) with viral loads (Figure 2B). Concurrently, pre-F-IgG titers post-second immunization in the MF59+pre-F, Alum+pre-F, and pre-F alone groups elicited the highest titers, with no significant difference between them (*p* > 0.05; Figure 2C). The Alum+pre-F group had significantly higher IgG titers than the CpG+pre-F group (*p* < 0.05) and R848+pre-F group (*p* < 0.05) (Figure 2C). The IgG titer post-second immunization had a mediate negative correlation coefficient (r = −0.5036) with viral loads (Figure 2D). This indicated that the early binding of IgG was less related to protection efficacy until post-second immunization.

To evaluate whether adjuvant-driven Th1/Th2 immune polarization was linked with protection, pre-F-specific IgG1 (Th2-associated) and IgG2a (Th1-associated) titers were measured post-second immunization. MF59+pre-F and Alum+pre-F induced comparable IgG1 titers, both numerically exceeding CpG, R848-adjuvanted groups, and pre-F-alone groups (*p* > 0.05; Figure 2E). In contrast, CpG+pre-F induced significantly higher IgG2a titers than the Alum+pre-F and R848+pre-F groups (Figure 2F). CpG-, R848-, and MF59-adjuvanted groups exhibited lower IgG1/IgG2a ratios than pre-F-alone, confirming Th1 bias. Alum exhibited a markedly elevated ratio compared to pre-F-alone, indicating dominant Th2 bias (Figure 2G). However, whatever IgG1 titer, IgG2a titer, or the ratios, they had low correlation coefficients (r = −0.3558, r = 0.1717, or r = −0.1205) with viral loads (Figure 2H and Figure S1A,B). Collectively, neither IgG1, IgG2a, nor their ratio correlated with protection, excluding Th1/Th2 polarization as a predictive biomarker.

### 3.3. Innate Immune Cell Responses Do Not Correlate with Protection

**Figure 3:**
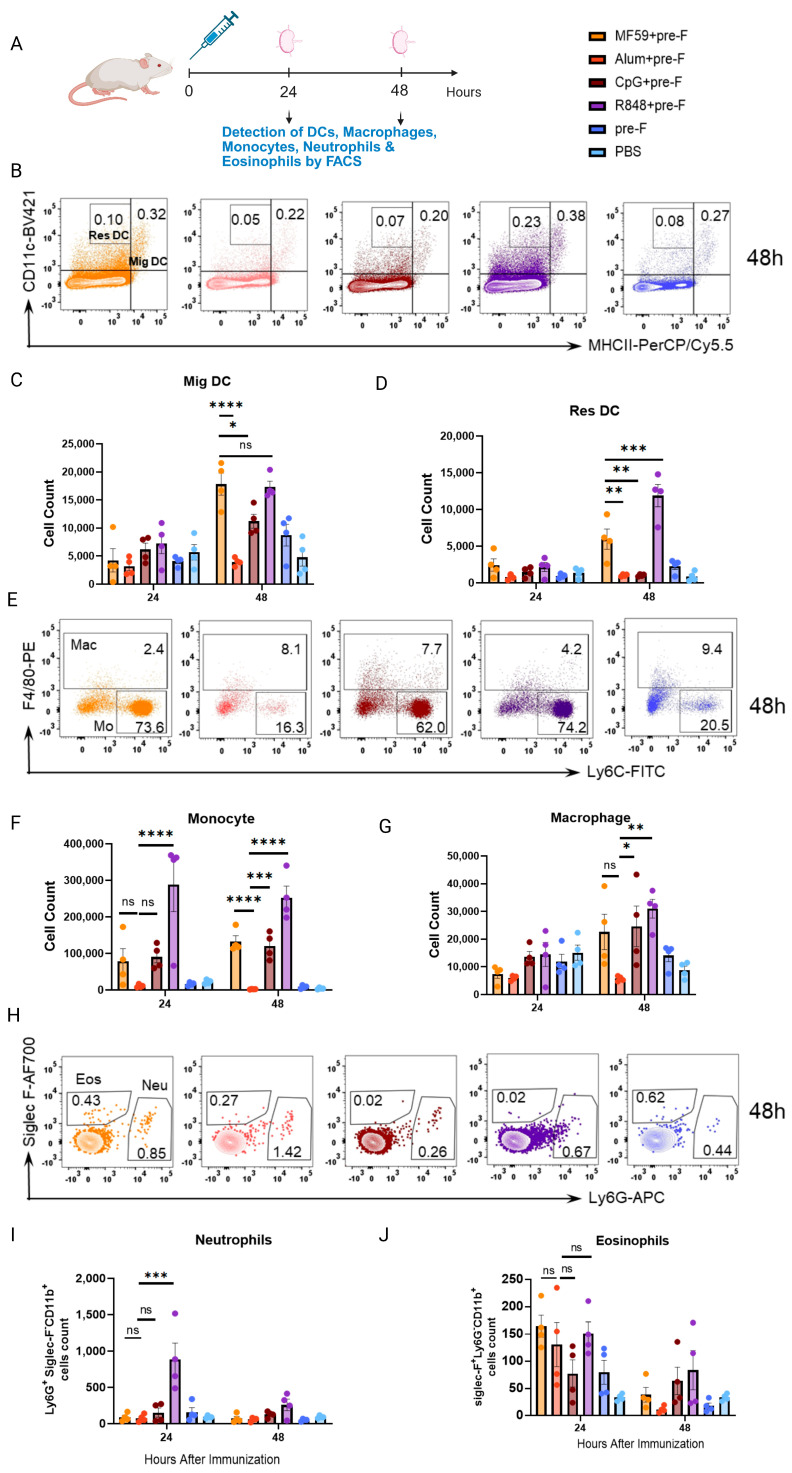
**Innate immune cell aggregation does not correlate with protective outcome.** Balb/c mice were immunized subcutaneously with various adjuvants and 10 µg of pre-F trimer. Following immunization, the mice were euthanized, and dLNs were collected at 24 h and 48 h post-immunization. Various cell types, including Mig DCs, Res DCs, Macrophages, Monocytes, Neutrophils, and Eosinophils, were analyzed. (**A**) Experiment schematic for (**B**–**J**). The orange dots: MF59+pre−F; The red dots: Alum+pre−F; The brown dots: CpG+pre−F; The purple dots: R848+pre−F; The blue dots: pre−F alone; The light blue dots: PBS control. (**B**) Representative flow plots of Res DCs and Mig DCs in draining LN. Live, single cells were gated on CD11c^hi^ MHC-II^mid^ for Res DCs and CD11c^+^ MHC-II^hi^ for Mig DCs. Quantification of Res DCs (**C**) and Mig DCs (**D**). (**E**) Representative flow plots of macrophages and monocytes in draining LN. Live, single cells were gated on CD11b^+^Siglec-F^-^Ly6G ^−^F4/80^+^ for macrophages and CD11b^+^Siglec-F^-^Ly6G^-^F4/80^−^Ly6C^+^ for monocytes. Quantification of macrophages (**F**) and monocytes (**G**). (**H**) Representative flow plots of neutrophils and eosinophils in draining LN. Live, single cells were gated on CD11b^+^Ly6G^+^Siglec-F^−^ for neutrophils and CD11b^+^Ly6G^−^Siglec-F^+^ for eosinophils. Quantification of neutrophils (**I**) and eosinophils (**J**). Data shown are means ± SEM from two independent experiments (n = four mice/group). **** *p* < 0.0001; *** *p* < 0.001; ** *p* < 0.01; * *p* < 0.05; ns (not significant) by one-way ANOVA.

Innate cells are primary targets of adjuvant activity. To investigate whether innate immune cells that activate Tfh cell differentiation correlate with vaccine-induced protection, we quantified dendritic cells (DCs), macrophages, monocytes, neutrophils, and eosinophils in mice at 24 h and 48 h post-primary immunization (Figure 3A).

Dendritic cells were categorized by origin: migratory Dendritic cells (Mig DCs; CD11c^+^ MHC-II^hi^), which are tissue-derived antigen-presenting cells (APCs) recruited to draining lymph nodes (dLNs), and resident dendritic cells (Res DCs; CD11c^hi^ MHC-II^mid^), which are LN-resident or blood-derived (Figure S2). Flow cytometry revealed that all adjuvanted groups except Alum+pre-F increased Mig DCs counts at 48 h versus 24 h (Figure 3B,C). MF59+pre-F and R848+pre-F induced comparable Mig DCs levels (*p* > 0.05), and MF59+pre-F was significantly higher than Alum+pre-F (*p* < 0.0001) and CpG+pre-F (*p* < 0.05). For Res DCs, R848+preF induced the strongest response among other adjuvanted groups (vs MF59+preF, *p* < 0.001), while MF59+preF exceeded Alum+pre-F (*p* < 0.01) and CpG+pre-F (*p* < 0.01) at 48 h (Figure 3D). This indicated that the abundance of Mig DCs and Res DCs did not correlate with challenge protection.

Monocytes were defined as CD11b^+^Siglec-F^-^Ly6G^-^F4/80^−^Ly6C^+^ and macrophages as CD11b^+^Siglec-F^-^Ly6G ^−^F4/80^+^ cells (Figure S3). Flow cytometry analysis revealed that all adjuvanted groups, except Alum+pre-F, induced a significant increase in monocyte counts at both 24 h and 48 h post-immunization (Figure 3E,F). Similarly, macrophage counts were significantly elevated by all adjuvants except Alum+pre-F (Figure 3E,G). Critically, the magnitude of monocyte and macrophage recruitment did not correlate with challenge protection outcomes.

Neutrophils (CD11b^+^Ly6G^+^Siglec-F^−^) showed a transient increase only in the R848+pre-F group at 24 h, which rapidly declined to baseline levels by 48 h; no significant changes were observed in other groups over this period (Figure 3H,I). In contrast, eosinophils (CD11b^+^Ly6G^−^Siglec-F^+^) were significantly increased by all adjuvanted groups compared to controls at 24 h and rapidly declined by 48 h. Alum+pre-F was no different to MF59+pre-F and R848+pre-F (Figure 3J).

Collectively, these results indicate that adjuvant-driven aggregation of innate immune cells—including DCs, macrophages, monocytes, and neutrophils—did not align with neutralizing antibody titers or protection against viral challenge. Specifically, Alum-adjuvanted formulations induced minimal innate immune cell aggregation compared to MF59 or TLR adjuvants.

### 3.4. Early Germinal Center B Cell Responses Correlate with Vaccine−Mediated Protection

**Figure 4:**
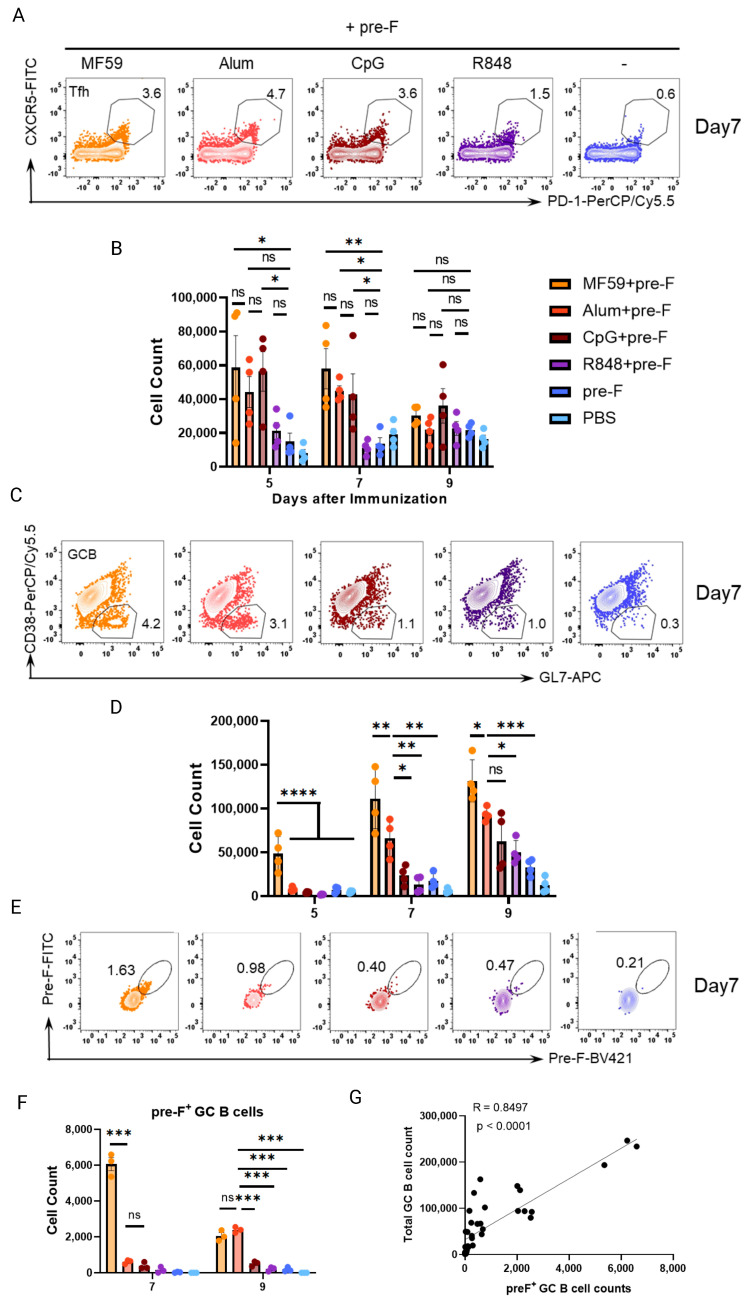
**Adjuvant**−**induced GC B cell responses align with enhanced protection.** Balb/c mice were immunized subcutaneously with 10 μg pre-F trimer and various adjuvants. dLNs were harvested at days 5, 7, and 9 post−immunization for flow cytometry. The orange dots: MF59+pre−F; The red dots: Alum+pre−F; The brown dots: CpG+pre−F; The purple dots: R848+pre−F; The blue dots: pre−F alone; The light blue dots: PBS control. (**A**,**B**) Representative flow plots (**A**) and quantification (**B**) of CD3^+^CD4^+^PD-1^+^CXCR5^+^ Tfh cells in dLN. (**C**,**D**) Representative flow plots (**C**) and quantification (**D**) of B220^+^GL7^+^CD38^-^ GCB cells in dLN. Representative data from two independent experiments, each with four mice per group (means ± SEM). (**E**,**F**) Representative flow plots (**E**) and quantification (**F**) of pre-F-specific GC B cells in draining LN at days 7 and 9 post-immunization. (**G**) Pearson correlation between the pre-F-specific GC B cells and total GC B cells. Representative data from two independent experiments, each with three mice per group (in (**E**–**G**); means ± SEM). **** *p* < 0.0001; *** *p* < 0.001; ** *p* < 0.01; * *p* < 0.05; ns (not significant) by one-way ANOVA.

Tfh cells are the key regulators for the activation of B cells. To understand the Tfh expansion in the early stage and its potential link to protection, the absolute numbers of Tfh cells were measured from day 5 to day 9 following immunization (Figure 4A,B). On days 5 and 7, the groups receiving MF59, Alum, and CpG adjuvants had a greater number of Tfh cells compared to pre-F alone, with no significant differences observed among the MF59, Alum, and CpG adjuvanted groups. By day 9, Tfh populations declined across all groups, with no significant differences compared to the pre-F alone (*p* > 0.05). These data demonstrate adjuvant-dependent Tfh expansion; however, the dynamic of Tfh in the early stage is less aligned with the efficacy enhanced by adjuvants.

GC B cell responses are critical for antibody class-switching and high-affinity antibody production. To investigate the expansion of GC B cells in the early stage and its potential connection to protective immunity, adjuvant-driven GC B cell kinetics were analyzed post-immunization (Figure 4C,D). At day 5, MF59+pre-F showed an early trend of GC B cell elevation compared to all of groups including Alum+pre-F, CpG+pre-F, or R848+pre-F (*p* < 0.0001). By day 7, MF59+pre-F generated significantly higher GC B cell counts than Alum+pre-F, while Alum+pre-F itself induced responses exceeding those of CpG+pre-F (*p* < 0.05), R848+pre-F (*p* < 0.01), and pre-F alone (*p* < 0.01). This trend persisted at day 9, with MF59+pre-F sustaining superior GC B cell numbers over Alum+pre-F (*p* < 0.05), and Alum+pre-F maintaining significantly greater responses than R848+pre-F (*p* < 0.05) and pre-F alone (*p* < 0.001), comparable with CpG+pre-F (*p* > 0.05). Notably, GC B cell expansion at days 7–9 was aligned with both neutralizing antibody titers and vaccine protection across adjuvanted groups. However, Alum+pre-F achieved comparable protection efficacy, despite eliciting significantly lower GC B cell responses than MF59+pre-F (*p* < 0.05 at day 9).

Next, to determinate the quantity of antigen-specific GC B cells following the increasing numbers of total GC B cells, we measured the pre-F specific GC B cells on day 7 and day 9 following immunization. Flow cytometric analysis revealed that MF59+pre-F elicited the significantly largest population of pre-F^+^ GC B cells among all groups at day 7 post-immunization (*p* < 0.001 vs. other adjuvants). By day 9, Alum+pre-F exhibited a marked increase in pre-F^+^ GC B cells, reaching levels comparable to the MF59+pre-F group (*p* > 0.05), significantly higher than other adjuvanted groups (*p* < 0.001 vs. other adjuvants) (Figure 4E,F). The specific GC B cells were tightly correlated with the number of total GC B cells (r = 0.8497) (Figure 4G), which indicated that the quantity of total GC B cells at day 7 and day 9 post-immunization was a key biomarker for the prediction of efficacy.

Plasma cells, as antibody-secreting effector cells, were quantified to assess their temporal association with vaccine-induced immunity (Figure S4). On day 5 post-immunization, MF59-adjuvanted groups exhibited increased plasma cell numbers compared to baseline, though no statistically significant differences were observed among adjuvants (*p* > 0.05). On day 7, MF59, Alum, and CpG further elevated plasma cell counts, yet comparisons with Alum+pre-F remained nonsignificant (MF59 vs. Alum and CpG: *p* > 0.05). On day 9, Alum+pre-F demonstrated no significant plasma cell expansion relative to R848+pre-F, MF59+pre-F, CpG+pre-F, or pre-F alone groups (*p* > 0.05).These findings confirm that early plasma cell expansion, while adjuvant-modulated, is not predictive of vaccine protection, whereas GC B cell dynamics serve as critical biomarkers of humoral immunity.

### 3.5. Follicular Dendritic Cell (FDC) Abundance Correlates with GC B Cell Response to Predict Protection

**Figure 5:**
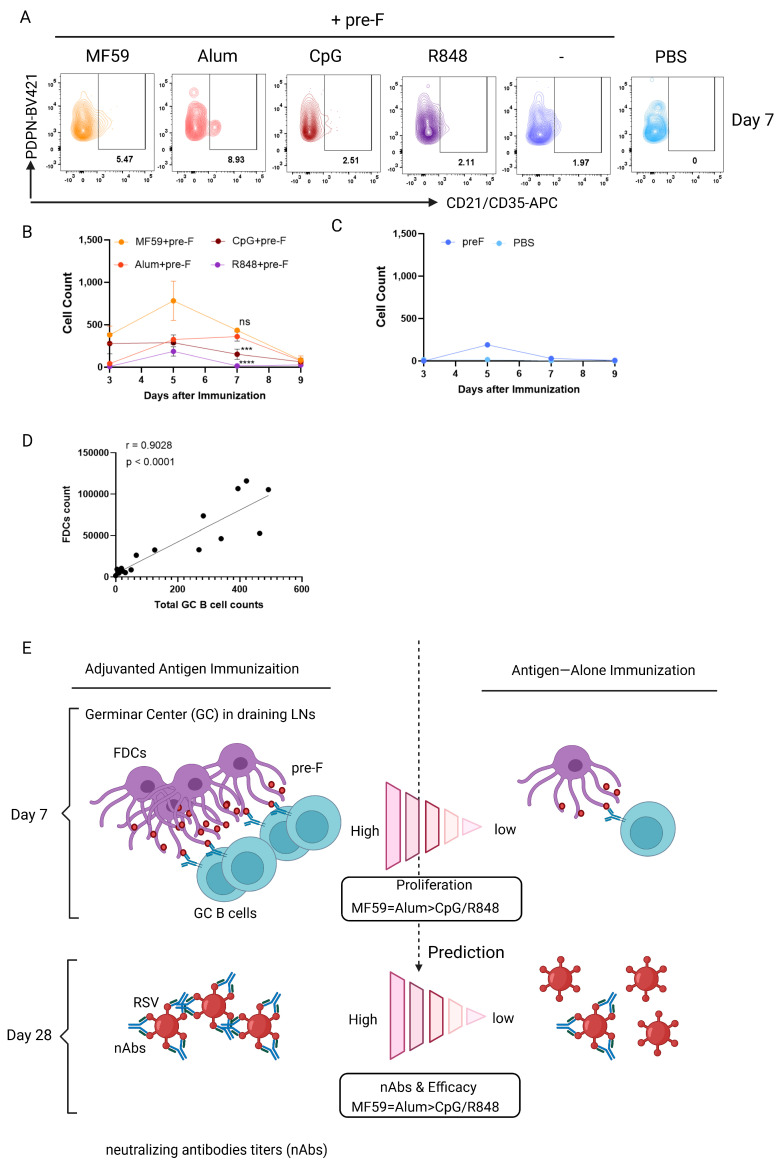
**Follicular dendritic cell (FDC) abundance correlates with GC B cell responses.** Balb/c mice were immunized subcutaneously with various adjuvants and 10 ug of pre-F trimer and euthanized at days 3, 5, 7, and 9 to isolate dLNs, respectively. FDCs were analyzed by flow cytometry. Representative flow plots (**A**) and quantification (**B**,**C**) of FDCs in draining LN. Live single cells were gated on CD45^-^B220^-^CD31^-^PDPN^+^CD21/CD35^+^ for FDCs (the gating strategy is shown in Supplementary Figure S3). (**D**) Pearson correlation between the FDCs and total GC B cells. (**E**) Schematic illustrating the prediction of adjuvant-elicited neutralizing antibody efficacy based on early germinal center responses. Data shown are means ± SEM from two independent experiments (n = 3 mice/group). **** *p* < 0.0001; *** *p* < 0.001; ns (not significant) by one-way ANOVA.

FDCs play a pivotal role in the humoral immune response by serving as the critical stromal platform for GC formation. To investigate the expansion of FDCs in the early stage and its potential link to protection, the FDCs’ kinetics were tracked in dLNs from days 3 to 9 post-single-dose immunization. FDCs were rigorously defined using FACS as CD45^−^ (excluding immune cells), B220^−^ (excluding B cells), CD31^−^ (excluding endothelial cells), PDPN^+^ (podoplanin, further defining follicular stromal cells including FDCs), and CD21/CD35^+^ (complement receptor 1 and 2, a marker of mature FDCs) (Figure S5). MF59 + pre-F induced a robust and rapid expansion of the FDC network. Significantly elevated FDCs counts were evident as early as day 3 and were sustained at the highest levels among all groups continuously through day 7. In contrast, the Alum + pre-F formulation elicited a slower initial increase in FDC abundance compared to MF59 + pre-F. However, this response reached a significantly higher peak FDC expansion, specifically at day 7, when compared to the CpG + pre-F (*p* < 0.001) or R848 + pre-F formulations (*p* < 0.0001), with no significant difference compared to MF59 + pre-F (*p* > 0.05). On the other hand, formulations containing CpG or R848, as well as the pre-F alone, induced earlier increases in FDC counts; however, these increases were followed by a rapid decrease (Figure 5A–C). This suggests a limited capacity to drive a robust antigen-retaining FDC network sufficient for optimal GC progression. Critically, day 7 FDC abundance strongly correlated with GC B cell counts (r = 0.9028) (Figure 5D) and universally predicted protection across adjuvants, including Alum. This establishes day 7 FDC abundance as a predictive biomarker that is independent of and complementary to GC B cells to resolve Alum’s prediction gap.

Collectively, the adjuvanted groups exhibited elevated FDC counts in dLNs, aligning with both GC response and the titers of neutralizing antibodies against viral challenge. The early abundance of FDCs and GC B cells serves a complementary and independent predictor of the magnitude of humoral immune response and vaccine protective efficacy. The strong proliferation of FDCs and GC B cell observed on days 7 post-prime immunization is aligned with elevated neutralizing antibody titers at later stages following the boost immunization on day 28. These early cellular metrics may serve as prognostic indicators of vaccine-induced protection (Figure 5E).

## 4. Discussion

This study establishes FDCs abundance and germinal center (GC) B cell responses as complementary early biomarkers for rapid prediction of vaccine efficacy in adjuvant screening. While GC B cell responses at days 7–9 post-immunization robustly correlated with protection across most adjuvants, Alum constituted a critical exception—eliciting robust protection despite significantly lower GC B cell activation than MF59. Crucially, FDC network maturation at day 7 universally predicted protective efficacy across all adjuvants tested, including Alum. By replacing prolonged serological-/challenge-based assessments (4–8 weeks/cycle) with day 7 FDC and GC B cell profiling, this paradigm shift accelerates preclinical adjuvant optimization and enables rational design of next-generation vaccines targeting FDC network maturation.

The recently approved RSV vaccine, Arexvy, utilizes the AS01E adjuvant system, which is a liposomal formulation containing saponin QS-21 and 3-O-desacyl-4′-monophosphoryl lipid A (MPL) [27]. In the present study, we employed MF59, a squalene-based oil-in-water emulsion, as a well-characterized benchmark adjuvant to evaluate the immunogenicity of our candidate antigen. Future studies will be necessary to directly compare different adjuvant platforms, including AS01E.

Based on the findings of this study, antigen-specific binding IgG titers measured after prime immunization do not correlate with protection efficacy or neutralizing antibody (nAb) levels until at least two weeks post-boost. This delay arises from low-affinity IgG during the prime phase, as affinity maturation occurs later in the GC reaction. While Th2-biased adjuvants (e.g., Alum) are traditionally associated with robust humoral responses, Th1-biased adjuvants (e.g., MF59) elicited comparable nAb titers and protection outcomes. Critically, Th1/Th2 bias alone proved insufficient as a predictor of immune efficacy, underscoring the need for multifactorial evaluation frameworks. While systemic vaccination is the current route used for RSV vaccines, mucosal vaccination would mimic RSV infection and promotes the production of antigen-specific IgA. Future studies will need to address development of adjuvanted mucosal vaccination.

GC reactions are indispensable for generating high-affinity, protective antibodies, with Tfh cells orchestrating B cell differentiation through cognate interactions involving CD40-CD40L, ICOS-ICOSL, and IL-21 signaling [28,29,30]. Our data reveal a critical dissociation between Tfh cell expansion and functional GC outcomes in adjuvanted vaccine contexts. While CpG-adjuvanted pre-F induced Tfh expansion comparable to MF59 and Alum formulations, this Tfh response failed to enhance GC B cell activity or generate pre-F -specific GC B cells. CpG’s failure to convert Tfh expansion into GC activity—despite recruiting DCs/monocytes—may stem from excessive IFN-α/IL-12 from CpG-activated DCs, which could skew Tfh away from IL-21-dominated GC-promoting phenotypes [31,32]. In addition to Tfh cells, FDCs are also essential for the proliferation of GC B cells. On day 7, the number of FDCs in the CpG-adjuvanted group was significantly lower than that in the MF59 and Alum adjuvant groups (Figure 5B). This finding suggests that the CpG adjuvant does not fully promote an effective GC response, likely due to inadequate proliferation of FDCs. This dissociation reveals that Tfh quantity alone is insufficient to predict GC output or efficacy, challenging models prioritizing Tfh expansion as a surrogate endpoint.

Tfh differentiation relies on APC-mediated priming and sustained antigen presentation [18,33]. However, we observed no universal innate cell recruitment pattern across adjuvants. CpG, R848, and MF59 triggered rapid recruitment of DCs, monocytes, and macrophages to dLNs, aligning with their roles in antigen transport and T cell priming. Despite minimal innate cell recruitment to dLNs, Alum still supported robust GC B cell responses and Tfh function, suggesting alternative mechanism via stromal cell activation.

Our study reveals that FDC expansion, detectable as early as day 3 post-primary immunization with adjuvanted antigens, serves as a pivotal mechanism for germinal center (GC) establishment. This early FDC expansion—previously underreported in adjuvant studies—facilitates sustained antigen deposition on FDCs, mediated by upregulated CR1/2 expression, which enhances antigen retention beyond initial immune activation [23,34]. Critically, FDC kinetics exhibit adjuvant-specific patterns; prolonged FDC expansion observed with Alum or MF59 adjuvants correlates with robust humoral immunity, whereas CpG or R848 adjuvants induce rapid FDC contraction, leading to diminished antigen deposition. Additionally, Alum-activated FDCs maintain their abundance longer than those activated by CpG or R848. This prolonged presence enhanced antigen retention, which is linked to increased proliferation of GC B cells. In future studies, we aim to investigate the mechanisms by which Alum enhances FDC antigen retention. These findings position FDC dynamics as a novel biomarker for early vaccine efficacy assessment, underscoring the importance of adjuvant selection in modulating FDC networks for durable immune responses.

Our study identifies the expansion of FDCs on day 7 post-primary immunization as an early predictor of protective immunity, correlating significantly with antigen deposition magnitude. However, technical limitations persist due to inconsistent definitions of FDC markers in the literature [23,35]. PDPN and CD21/CD35 are broadly accepted FDC markers, which are known to play critical roles in FDC functions. A lack of PDPN and CD21/CD35 results in a diminished GC response [23,35,36,37]. Consequently, these two markers are standardized across laboratories. However, further characterization of subsets within the FDC population is necessary for a more precise definition. We will continue to investigate the roles of additional markers, such as EpCAM and Madcam1, to better elucidate FDC heterogeneity and function. Whether these newly reported markers offer more precise functional definitions of FDCs or identify specific subsets warrants rigorous experimental validation in future studies. This variability underscores the need for deeper exploration of FDC functional characteristics. We propose that FDCs’ role as predictors hinges on abundance, which synchronize with germinal center B cell responses to enable sustained affinity maturation. Resolving these technical ambiguities will refine FDC-based early efficacy assessments and reveal new targets for vaccine design.

This study represents a significant step toward accelerating vaccine adjuvant screening by identifying early FDC and GC B cell responses as predictive biomarkers. However, its limitations—limited adjuvant scope, reliance on a single mouse model, short-term focus, FDC marker variability, small sample sizes, and mechanistic gaps—suggest caution in interpreting its broader applicability. We will explore newer platforms, such as nanoparticles and STING agonists, as potential strategies to enhance the universality of the biomarker. Future research addressing these issues will be essential to validate and refine these biomarkers, ultimately supporting the development of more effective and rapidly deployable vaccines.

## 5. Conclusions

In summary, this study suggests that FDC abundance and GC B-cell responses could provide supportive, early indicators of vaccine efficacy during preclinical adjuvant screening at days 7–9 post-immunization. While further validation—ideally with additional adjuvants—is needed, this approach may offer a modest step toward streamlining adjuvant selection and informing vaccine design. When facing a novel influenza strain or X-virus, this method allows for the evaluation and selection of the most effective vaccine adjuvant within just 7–9 days as opposed to the weeks or months typically required. Such rapid screening enables quicker advancement of candidate vaccines into clinical trials and large-scale production, ultimately accelerating the availability of effective vaccines.

## Figures and Tables

**Table 1:** Experimental groups and composition.

Groups	pre-F/Mouse	Adjuvant/Mouse	Volume/Mouse
PBS	/	/	100 μL
pre-F	10 μg	/	100 μL
pre-F+MF59	10 μg	MF59 50 μL	100 μL
pre-F+Alum	10 μg	Alum 100 μg	100 μL
pre-F+CpG	10 μg	CpG 20 μg	100 μL
pre-F+R848	10 μg	R848 20 μg	100 μL

## Data Availability

All data reported in this paper will be shared by contacting the lead contact upon request. Any additional information required to reanalyze the data reported in this work is available from the lead contact upon request.

## Supplementary Materials

The following supporting information can be downloaded at https://www.mdpi.com/article/10.3390/vaccines13101011/s1: Figure S1: The second IgG2a titer and the IgG1/IgG2a ratio exhibited a lower correlation with protection; Figure S2: The FACS gating strategy used to identify single live CD11c^hi^ MHCII^med^ Res DCs and CD11c^+^MHCII^hi^ Mig DCs; Figure S3: The FACS gating strategy used to identify single live eosinophils (Eos), neutrophils (Neu), macrophages (Macro), and monocytes; Figure S4: The dynamic of plasma cells enhanced by varying adjuvants; Figure S5: The FACS gating strategy used to identify single live CD45^-^B220^-^CD31^-^PDPN^+^CD21/CD35^+^ FDCs.
