# Supplementary material for: Accelerating Vaccine Adjuvant Screening: Early Follicular Dendritic Cell and Germinal Center B Cell Biomarkers Predict Protective Efficacy
Source: Vaccines (Basel). 2025 Sep 28;13(10):1011. doi: 10.3390/vaccines13101011 (PMC12567668; doi:10.3390/vaccines13101011)
Supplement: Supplementary file 1 [file vaccines-13-01011-s001.zip › vaccines-3840783-supplementary.pdf]

Supplementary data

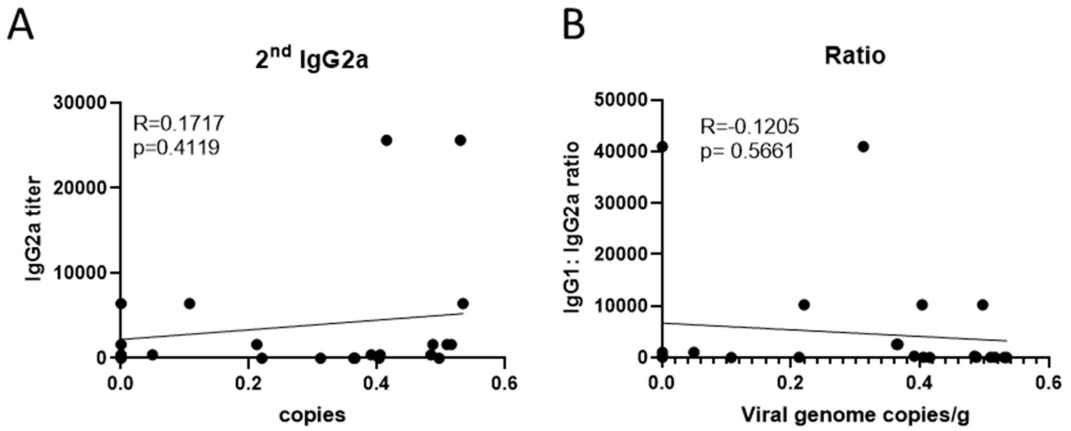

Supplementary Figure 1 the 2nd IgG2a titer and the IgG1/IgG2a ratio exhibited a lower correlation with protection. The data for the IgG2a titer and the IgG1/IgG2a ratio were obtained from Figures 2F and 2G. The correlation analysis for the IgG2a titer with protection is presented (A), while the correlation between the IgG1/IgG2a ratio and protection is illustrated (B).

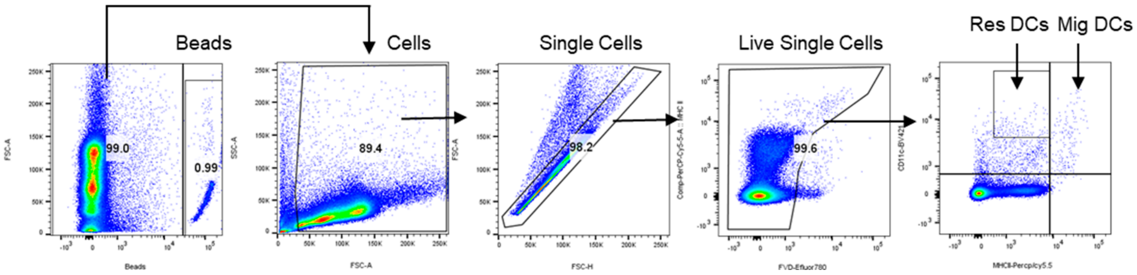

Supplementary Figure 2 the FACS gating strategy used to identify single live CD11c^{hi} MHCII^{med} Res DCs and CD11c^{+}MHCII^{hi} Mig DCs.

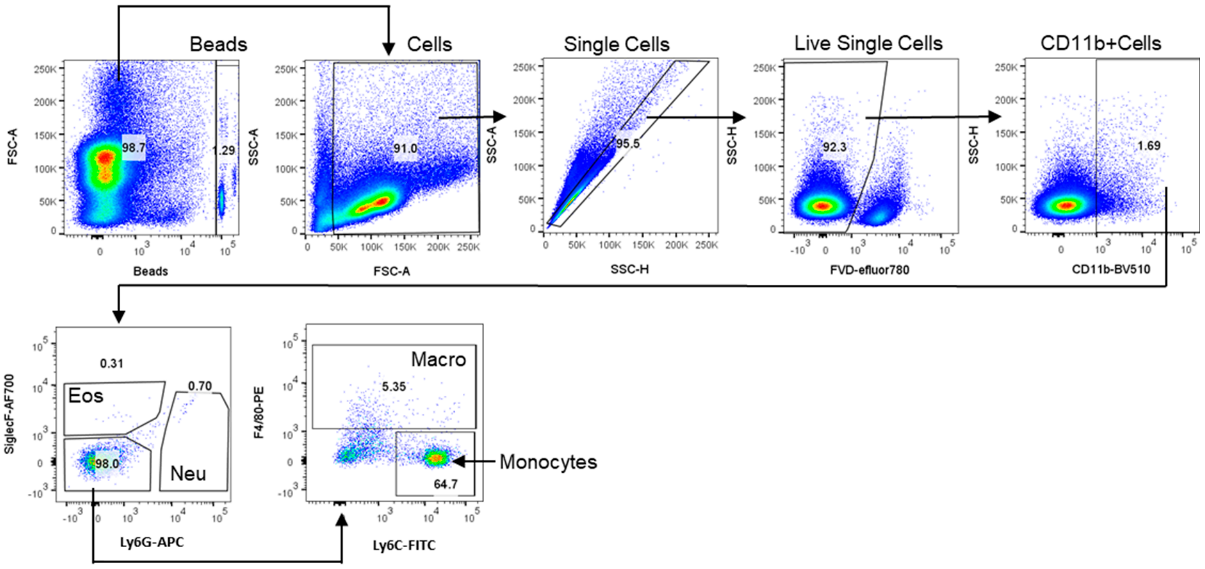

Supplementary Figure 3 the FACS gating strategy used to identify single live Eosinophils (Eos), Neutrophils (Neu), Macrophages (Macro), and Monocytes.

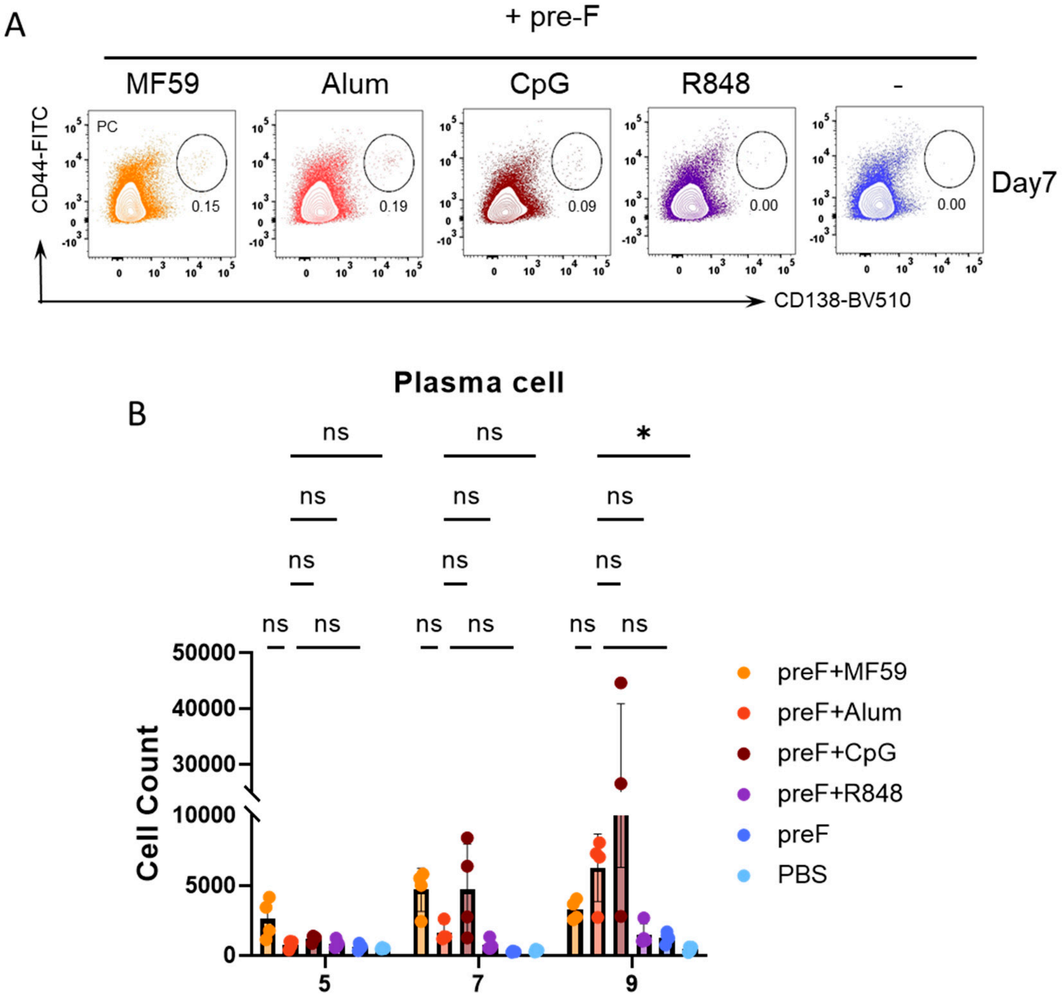

Supplementary Figure 4 the dynamic of plasma cells enhanced by varying adjuvants.

Balb/c mice were immunized subcutaneously with 10 μg pre-F trimer and various adjuvants. Draining LNs were harvested at Days 5, 7, and 9 post immunization for flow cytometry. (A-B) Representative flow plots (A) and quantification (B) of CD138^{+}CD44^{+} plasma cells in dLN.

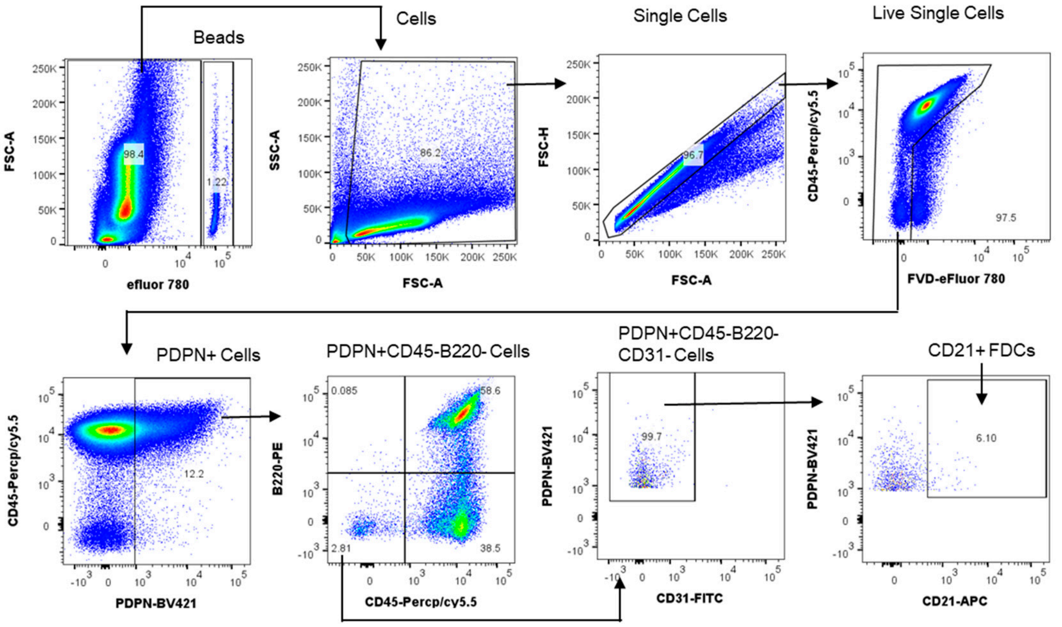

Supplementary Figure 5 the FACS gating strategy used to identify single live PDPN$^+$CD45$^-$B220$^-$CD31$^-$CD21$^+$ FDCs.
